# Synthesis and characterization of Mono-disperse Carbon Quantum Dots from Fennel Seeds: Photoluminescence analysis using Machine Learning

**DOI:** 10.1038/s41598-019-50397-5

**Published:** 2019-09-30

**Authors:** Akansha Dager, Takashi Uchida, Toru Maekawa, Masaru Tachibana

**Affiliations:** 1 Graduate School of Nanobioscience, Yokohama City University, 22-2 Seto, Kanazawa-ku, Yokohama, 236-0027 Japan; 3 Present Address: Bio-Nano Electronics Research Centre, Toyo University, 2100 Kujirai, Kawagoe, Saitama 350-8585 Japan; 2 Silicone-Electronics Materials Research Center, Shin-Etsu Chemical Co., Ltd., 1-10 Hitomi, Matsuida-machi, Annaka-shi, Gunma, 379-0224 Japan

**Keywords:** Materials science, Nanoscale materials, Quantum dots

## Abstract

Herein, we present the synthesis of mono-dispersed C-QDs via single-step thermal decomposition process using the fennel seeds (*Foeniculum vulgare*). As synthesized C-QDs have excellent colloidal, photo-stability, environmental stability (pH) and do not require any additional surface passivation step to improve the fluorescence. The C-QDs show excellent PL activity and excitation-independent emission. Synthesis of excitation-independent C-QDs, to the best of our knowledge, using natural carbon source via pyrolysis process has never been achieved before. The effect of reaction time and temperature on pyrolysis provides insight into the synthesis of C-QDs. We used Machine-learning techniques (ML) such as PCA, MCR-ALS, and NMF-ARD-SO in order to provide a plausible explanation for the origin of the PL mechanism of as-synthesized C-QDs. ML techniques are capable of handling and analyzing the large PL data-set, and institutively recommend the best excitation wavelength for PL analysis. Mono-disperse C-QDs are highly desirable and have a range of potential applications in bio-sensing, cellular imaging, LED, solar cell, supercapacitor, printing, and sensors.

## Introduction

Carbon quantum dots (C-QDs) have gained much attention due to their chemical stability, excellent water solubility, low cost, and fluorescence properties. Synthesis of C-QDs is roughly classified into “top-down” and “bottom-up” approaches^1,2^. In the top-down approach, C-QDs are synthesized by breaking the large carbon materials into smaller ones by employing the arc discharge^3^, laser ablation^4^, and chemical oxidation^5^. Conversely, in the bottom-up approach, C-QDs are synthesized via molecular carbon precursors using methods such as; hydrothermal^6^, thermal-decomposition^7^, and microwave^8–11^. Majority of the synthesis processes are typically multistep, cumbersome, and require expensive carbon sources that might be toxic at times, followed by the further necessity of surface passivation^12–15^.

There are many green synthesis routes have been devised for the synthesis of C-QDs by employing inexpensive and natural materials as starting carbon sources, such as chitosan^16^, egg yolk oil^15^, orange juice, lemon peel^16^, bee pollen^13^, collagen^14^, humic substances^17^, hair^18^, peanut shell^19^, soy milk^6^, Cashew Gum^20^ and Garlic^21^. However, most natural carbon sources used in these studies lack the homogeneity and essential purity to obtain the homogenous C-QDs^6,13,15–20^. The major hindrance with most of the natural carbon sources to synthesize the C-QDs is that they undergo seasonal fluctuation based on geographical location of plant cultivation that could undoubtedly influence reproducibility, shape and size distribution of C-QDs^12^. During the synthesis, the presence of a trace amount of foreign impurities (minerals) can lead to the inhomogeneous nucleation and growth of C-QDs. The selection of natural carbon source should be economical, reproducible, and environmental friendly. Also, the photoluminescence properties of as-synthesized C-QDs must be immutable to the sampling and sessional fluctuation. Hydrothermal or solvothermal methods with synthetic molecular carbon precursors (non-natural sources) provide more optional parameters that can felicitate the synthesis of nearly-monodisperse C-QDs^10,22^. On the contrary, using much straightforward thermal-decomposition (pyrolysis) process the control over the size distribution of C-QDs is extremely difficult to achieve given the complex chemical composition of the natural carbon sources^7,23^.

The fluorescence emission of the C-QDs is quite complex and not yet fully understood. Traditionally, pH-dependent photoluminescence (PL) spectra of C-QDs are analyzed at a fixed excitation and completely ignore many other possible excitations, however, using such approach only partial information is extracted^6,8,9,13,16,17,19–21,24,25^. Analyzing the C-QDs at different pH for a wide range of excitation can, additionally, provide much valuable information than the conventional techniques. However, an increase in the number of PL measurements can also increase the complexity of data analysis techniques. Recently, machine learning techniques such as k-means, principal component analysis (PCA), partial least squares analysis (PLS), hierarchical cluster analysis (HCA), non-negative matrix factorization (NMF), multivariate curve resolution (MCR-ALS) have accelerated the multivariate scientific data analysis^26^. Machine learning (ML) can classify even very similar PL spectra, and it is capable of handling large dataset (multiple-set PL measurements). Analyzing the hyper-spectral PL data-set of C-QDs with machine learning could potentially help to augment the origin of PL mechanism.

Herein, for the first time, we present the synthesis of mono-dispersed C-QDs via single-step thermal decomposition process using the fennel seeds (*Foeniculum vulgare*). Synthesis of C-QDs with narrow size distribution, to the best of our knowledge, using natural carbon source via pyrolysis process has never been achieved before. The effect of reaction time and temperature on pyrolysis provides insight into the synthesis of C-QDs. As synthesized C-QDs have excellent colloidal solubility, photo-stability, environmental stability (pH) and does not require any additional surface passivation step to improve the fluorescence. We have used the machine learning techniques (ML) to analyze the PL of as-synthesized C-QDs and try to address the following two issues; (i) is ML able to classify the multiple-set of pH-dependent PL measurements (PL spectra taken at various pH and excitation) and recommend the best excitation wavelength for comprehensive pH-dependent study? (ii) Can ML assist in finding out the source of PL mechanism given that the multiple-set PL measurements at various pH and excitation wavelength can activate the different type of surface state? C-QDs of narrow size distribution are highly desirable and have a range of potential applications in bio-sensing, cellular imaging, LED, solar cell^13,27,28^, supercapacitor^29^, printing^24,30^, and sensors^31^.

## Experimental Methods

### Materials

Fennel seeds (Swati seeds, India) were used without further purification. Deionized water (DI) was used throughout the experiment.

### Synthesis of C-QD

Carbon Quantum dots were synthesized by pyrolysis method. As received fennel seeds were crushed using a mixer grinder (Tiger mixer grinder, Japan). Ground greenish fennel powder (0.2 g) was transferred to the crucible cup (AS ONE, Japan) and was heated using a heat plate (AS ONE, Japan) at a constant temperature of 500 °C for 3 hours. Subsequently, the crucible was allowed to cool down to room temperature. Carbonization of the greenish fennel powder turned into a dark-gray product, and it was dissolved in deionized water followed by the sonication for 5 minutes. The black color suspension was centrifuged at 15000 rpm for 10 minutes to remove the large un-dissolved particles. The supernatant was filtered using 100 nm pore size filter (PALL ACRO DISC, Japan) and subjected to the dialysis using dialysis kit (Float-A-Lyzer G2 Dialysis, Japan) for further purification. The purified C-QDs was transferred to the glass vial and stored for further characterization.

### Characterization of C-QDs

The optical properties of C-QDs were studied using the UV-Vis absorption spectroscopy (V-530, JASCO, Japan) and photoluminescence spectroscopy (FP-6500, JASCO, Japan). The size distribution and structure of C-QDs were characterized by transmission electron microscopy (TEM, JEM2002-FS, JEOL). The surface charge and size distribution of C-QDs were estimated by Zeta-sizer (Nano-ZS 90 Zetasizer, Malvern Instruments Ltd). Elemental composition and structural purity of C-QDs were ascertained by energy-dispersive X-ray spectroscopy (EDS, JEM-2300F, JEOL) and laser Raman spectroscopy (Lab RAM, HR-800, Horiba JOBIN YVON S.A.S.), respectively. Fourier transform infrared spectra (FTIR) of dried C-QDs were recorded to identify the functional groups on to the surface of C-QDs (FTIR-4100, JASCO, Japan). The X-ray photoelectron spectroscopy (PHI Quantes, ULVAC-PHI. Inc.) analysis was done to ascertain the chemical state of as-synthesized C-QDs. The quantum yield of C-QDs was investigated by quantum efficiency measurement system (QE-2000, Otsuka Electronics, Japan). Thin-layer chromatography (TLC) analysis was done using TLC Silica gel 60 F254 plate (Merck Millipore) to ascertain the purity of the C-QDs.

### PL data-structure for ML

PL data was acquired using the excitations 200, 220, 240, 260, 280, 300, 320 and 340 nm, respectively for the following pH 3, 5, 7, 9, 11, 13. In total, forty-eight (48) PL measurements were taken, typically, single spectrum, i.e., intensity vs. wavelength is stored in a one-dimensional vector of length (*N* = 401, where *N* is the number of data points acquired in the spectral range 300–750 nm). Consequently, all the spectra were put together to form the two dimensional (2D) matrix; i.e., *D* having a dimension (48 × 401) containing the entire PL data set. Each row in the 2D data matrix represents one spectrum. Since the data have an excellent signal to noise ratio, no additional data processing was done. (See more details on PCA, MCR-ALS and Sparse NMF techniques in the supplementary information (SI)).

### ML techniques for PL data analysis

In order to have a plausible explanation for the origin PL mechanism. 2D data matrix containing PL data-set was analyzed by PCA, MCR, and sparse NMF (See more details in the supplementary information (SI)).

## Results and Discussion

**Figure 1:**
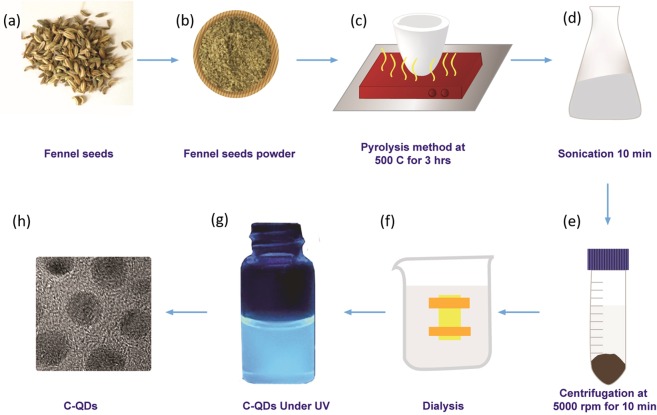
The schematic illustration shows the synthesis of C-QDs from fennel seed via pyrolysis method. (**a**) Fennel seeds, (**b**) ground fennel powder, (**c**) pyrolysis of fennel powder, (**d**) sonication of C-QDs, (**e**) centrifugation of C-QDs, (**f**) dialysis of C-QDs, (**g**) C-QDs under UV, and (**h**) TEM image of C-QDs.

**Figure 2:**
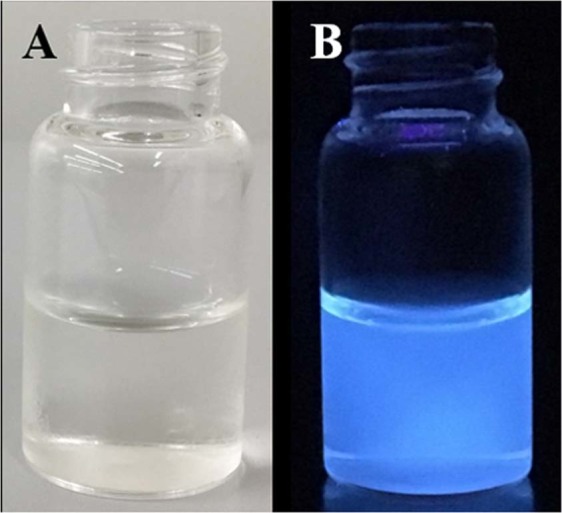
Optical images of C-QDs dispersed in water. (**A**) C-QDs under the normal daylight exposure, (**B**) C-QDs under the under UV exposure (365 nm).

**Figure 3:**
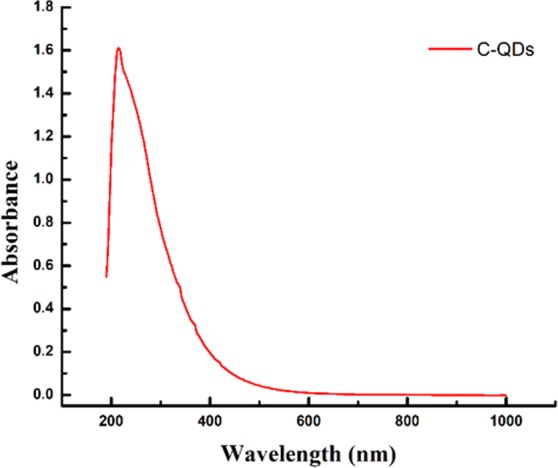
UV-Vis absorption spectra of as-synthesized C-QDs.

Fennel seeds are mainly composed of carbohydrates and possess lots of antimicrobial-cum-antioxidant properties, and that is the reason it is commonly cultivated and consumed around the world. Fennel seeds have never been used for the synthesis of carbon quantum dots (C-QDs). Carbon quantum dots were synthesized via pyrolysis process as illustrated in the Fig. 1. The optical image of C-QDs dispersed in water under normal light, and UV light is shown in Fig. 2a,b, respectively. The C-QDs suspended in water shows strong bluish fluorescence emission under UV exposure (365 nm). The absorption spectra of the as-prepared C-QDs is shown in Fig. 3, exhibit two peaks; a very strong peak centered at 220 nm, and another small shoulder peak at 338 nm peaks, attributed to the π- π* transition of C=C and n-π* transition of C=O bonds, respectively^8,17^.

**Figure 4:**
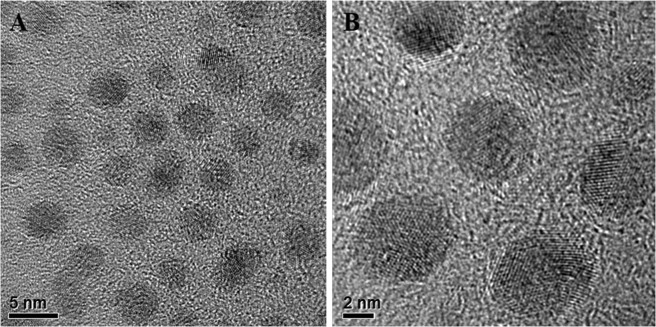
TEM images of as-synthesized C-QDs. (**A**) C-QDs at low resolution, (**B**) C-QDs at high resolution, crystalline lattices can be seen.

The low and high-resolution TEM images of C-QDs are shown in Fig. 4A,B, respectively. For the precise size distribution measurement, one hundred forty-eight (148) C-QDs nano-particles were taken into account, in total, forty-two high-resolution TEM images of C-QDs were recorded by scanning the random positions on to the TEM grid. The C-QDs were uniform in size and formed a hexagonal pattern, which indicates the mono-dispersity of as-synthesized C-QDs (see the Fig. 4B). C-QDs are highly mono-dispersed with the average size diameter of 3.90 ± 0.91 nm (see the SI Fig. S1 for the distribution of the diameters of C-QD). The lattice fringes within the particles indicate that the as-synthesized C-QDs were crystalline; noticing that the lattice spacing in the C-QDs was found to be 0.21 nm (see the SI Fig. S2 for high-resolution lattice spacing) can be assigned to (100) plane of carbon particle^24^. In general, C-QDs synthesized from natural and synthetic molecular carbon sources have the amorphous carbon structure; either the whole C-QD structure was amorphous, or C-QDs have a crystalline core with an amorphous outer shell^14,29^. However, in the present report, the structure of C-QDs was well crystalline, and it is because of the sufficient pyrolysis as confirmed by TEM images.

Unlike to the thermal decomposition process, the narrow size distribution of C-QDs can be easily achieved via hydrothermal method because molecular carbon precursors provide much needed homogenous nucleation to govern the size of as-synthesized C-QDs^24^. However, the narrow size distribution of C-QDs synthesized by thermal decomposition, in the present report, is comparable with hydrothermal method^24^ and it is quite remarkable because by using the natural carbon source such kind of narrow size distribution was never reported beforehand^14,17^. There are multiple reports where the authors have used the pyrolysis method and natural carbon source as starting materials; however, the synthesized C-QDs had a considerable size variation^16^. A point beam EDS analysis of carbon quantum dots shows that the C-QDs are consists of only carbon and oxygen peaks, indicating that the synthesized C-QDs have no elemental signature of any foreign impurities other than carbon and oxygen on the TEM grid (see the SI Fig. S3 for the point beam EDS of C-QD). The electron beam spot size was only a few nanometers and was directed on to a few C-QDs. However, it is indispensable to distinguish whether the carbon peak is originated from the C-QD sample or carbon peak is the superimposition of signals from the micro-grid supported carbon-film (TEM grid) and C-QDs. Nevertheless, the only objective of the EDS was only to ascertain that the C-QD sample on the grid does not contain any foreign residual element other than carbon and oxygen^12^.

**Figure 5:**
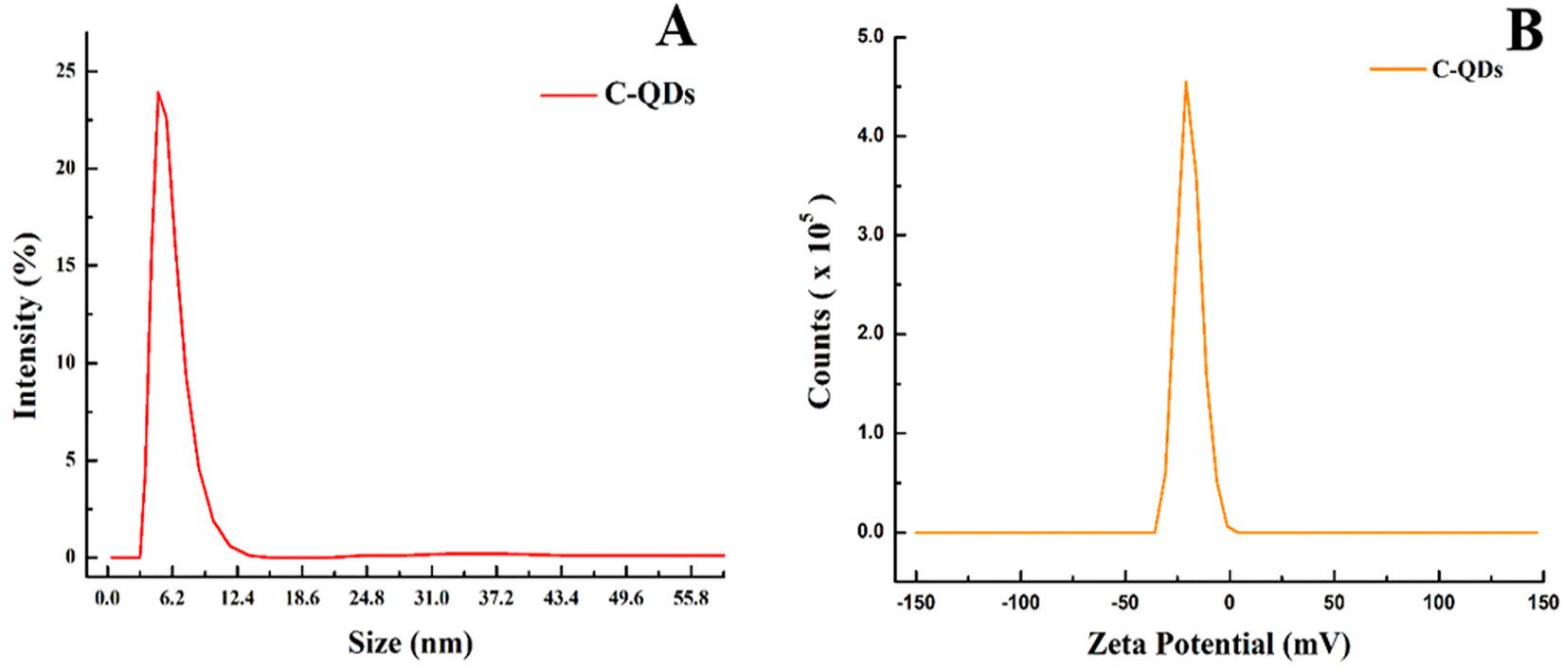
(**A)** Dynamic light scattering (DLS) size distribution curve of C-QDs dispersed in water, **(B)** Zeta-potential curve of C-QDs dispersed in water, shows C-QDs have a negative surface charge.

The size distribution of as synthesize C-QDs was verified by zeta-seizer, as shown in Fig. 5A. The average size of the C-QDs was found to be 6.1 nm, which is slightly larger than that was obtained with TEM; i.e., 3.90 nm. Note that the hydrodynamic diameter of a particle in the solvent is, in general, larger then the size of C-QDs measured in vacuum. Given the bare C-QDs are hydrophobic, C-QDs without any functional group on its surface can easily precipitate in the aqueous-based solvent. The existence of multiple functional groups over the C-QDs surface can improve the dispersion of C-QDs in a water-based solvent, that is why functionalization of C-QDs is the pre-requisite for a very stable colloidal stability over the long term^24,27^. The zeta-potential of C-QDs is shown in Fig. 5B, Zeta-potential of the C-QDs shows a single peak at negative 23 mV with a peak width of 10.01 mV. The negative potential indicates that the surface of C-QDs have negative charge moieties, such kind of moieties are essential to achieving a good dispersion of C-QDs in a water-based solvent. Storage of C-QDs at room temperature even after 15 month shows no sign of turbidity indicates that the colloidal stability of C-QDs is very high and can be used over a period of time (see the SI Fig. S4 of fresh C-QDs and C-QDs stored for 15 months)^29^.

**Figure 6:**
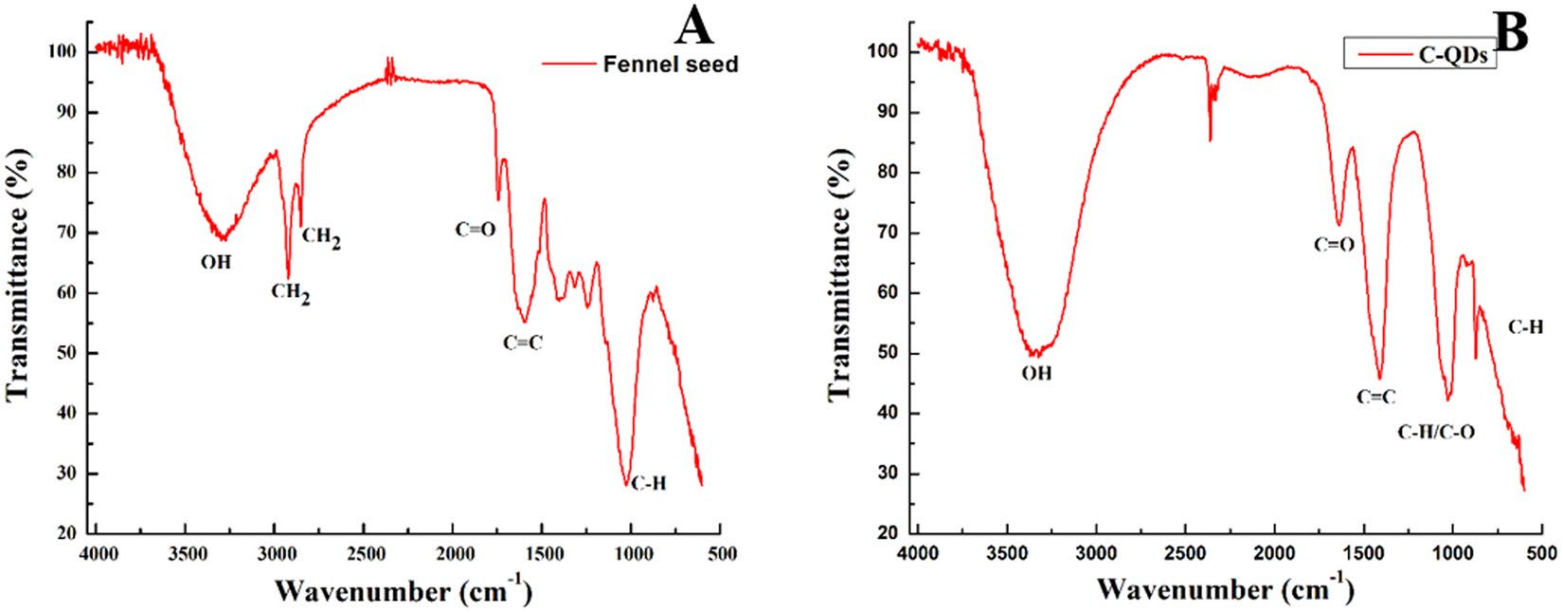
(**A)** FTIR spectra of ground Fennel Seeds (carbon precursor for the synthesis of C-QDs), **(B)** FTIR spectra of as-synthesized C-QDs. Carbon quantum dots have mainly the C=C, C=O and C-H peaks.

Although, the zeta potential of C-QDs confirms the presence of negative charge moieties on C-QDs surface, nevertheless, zeta potential alone cannot determine the chemical structure of moieties. FT-IR analysis of as prepared C-QDs helps to ascertain the possible chemical structure of the moieties attached on the surface of C-QDs. Moreover, we have also used FTIR analysis as an indicator to optimize the process parameters for the synthesis of C-QDs. The FT-IR spectrum of fennel seeds and as prepared C-QDs are shown in Fig. 6A,B, respectively. The FT-IR spectra of ground fennel powder have peaks at 3296, 2921, 2851, 1742, 1597 and 1027 cm^−1^ can be ascribed to vibrations OH stretching, symmetric CH_2_, asymmetric CH_2_, carboxyl/carbonyl C=O, C=C and C-H bending, respectively^29^. The symmetric and asymmetric CH_2_ peaks at 2921 and 2851 cm^−1^ in FTIR spectra of fennel powder indicate that the seeds were primarily composed of hydrocarbons^16^. On the other hand, FTIR spectra of C-QDs have significant peaks at 3350, 1640, 1405, 1023, 859 cm^−1^ can be ascribed to OH, C=O, C=C, (C-H or C-O), C-H bending, respectively^19,28^. The presence of C=C peak indicates that the C-QDs are made of graphitic structure, whereas the OH, C=O, and C-H bending peaks suggest that the C-QDs have OH, C=O and C-H surface moieties attached on its surface. FTIR and zeta-potential results complement each other and established the fact that the surface of as-synthesized C-QDs has intrinsic negatively charged moieties that are essential for long term water solubility^29^.

The absence of symmetric and asymmetric peaks of CH_2_ at 2921 and 2851 cm^−1^, respectively, in C-QDs FTIR spectra indicate that the carbon source (fennel seeds) was fully carbonized (hydrocarbon converted to the graphitic structure). At times, insufficient carbonization can lead to some of the functional group from the initial carbon sources to persist on the surface of as-synthesized C-QDs even after the pyrolysis; such C-QDs yield poor photoluminescence^32^. To avoid such kind of issues, in the present report, the peaks of CH_2_ were used as an indicator to optimize the process parameters such as temperature and time to synthesize well-graphitized C-QDs. Often, optimization of any synthesis process can be time-consuming and cumbersome, however, in the present case, it was straight forward, trials were done with temperature ranging from 200-, 300-, 400- to 500 °C for 1 to 5 hours. FT-IR results show that with an increase in temperature and time, the symmetric and asymmetric peaks of CH_2_ at 2921 and 2851 cm^−1^ gradually started to decrease and eventually vanished (see the SI Fig. S5 for FT-IR of one of the trials run to synthesize C-QD). The fennel seed sample was fully carbonized at 500 °C for 3 hours, and keeping it longer has not resulted in any significant change in either PL or crystallinity.

**Figure 7:**
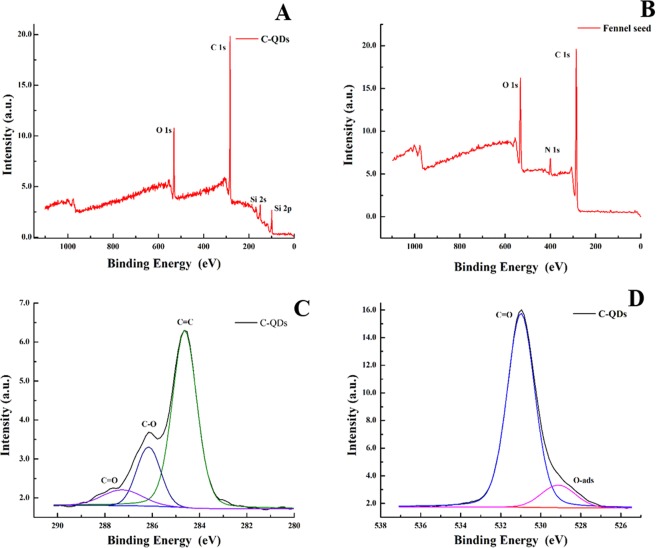
(**A)** XPS spectra of as-synthesized C-QDs (wide scan) shows carbon and oxygen peaks, **(B)** XPS spectra of ground fennel powder (wide scan) depicts that the seeds are mainly composed of carbon, nitrogen, and oxygen, **(C)** XPS spectra of C 1 s spectrum of as-synthesized C-QDs, spectrum was de-convoluted into three significant peaks at 284.6 (C=C), 286.16 (C-O) and 287.2 eV (C=O), respectively, **(D)** XPS spectra of O 1 s spectrum of as-synthesized C-QDs, spectrum was de-convoluted into two significant peaks at 529.13 (phy-Adsorb) and 531.1 eV (isolated-OH/C=O/O-C=O) respectively.

XPS analysis of as-synthesized C-QDs and ground fennel power is shown in Fig. 7. The survey scan of C-QDs XPS in Fig. 7A shows that the C-QDs are mainly composed of carbon and oxygen; i.e., C1s (71.2 at. %) and O 1s (28.8 at. %), respectively (see the SI Table S1). No other trace of any other residual element was detected which highlight the purity of the C-QDs. The survey scan of ground fennel powder shows that carbon source is made of carbon, nitrogen, oxygen (see Fig. 7B). Deconvolution of C1s peak is shown in Fig. 7C, the main peak at 284.6 eV (70.2 at. %) corresponds to sp^2^ graphitic structure, whereas the peaks at 286.16 (20.6 at. %) and 287.2 eV (9.2 at. %) are attributed to C-O and C=O, respectively (see the SI Table S2). One out of every four carbon atoms were functionalized with oxygen that shows the C-QDs have a high degree of functionalization. XPS results complement the FT-IR results, there too mainly C-O and C=O bond were detected). Deconvolution of oxygen peak is shown in Fig. 7D, peak at 529.13 (11.5 at. %) is attributed to physically adsorbed oxygen on the surface of C-QDs, whereas the peak at 531.1 eV (88.5 at. %) are originated from functional groups; i.e., isolated-OH/C=O/O-C=O (see the SI Table S3)^33^. The high degree of functionalization is the main reason for C-QDs to possess long-term colloidal stability.

**Figure 8:**
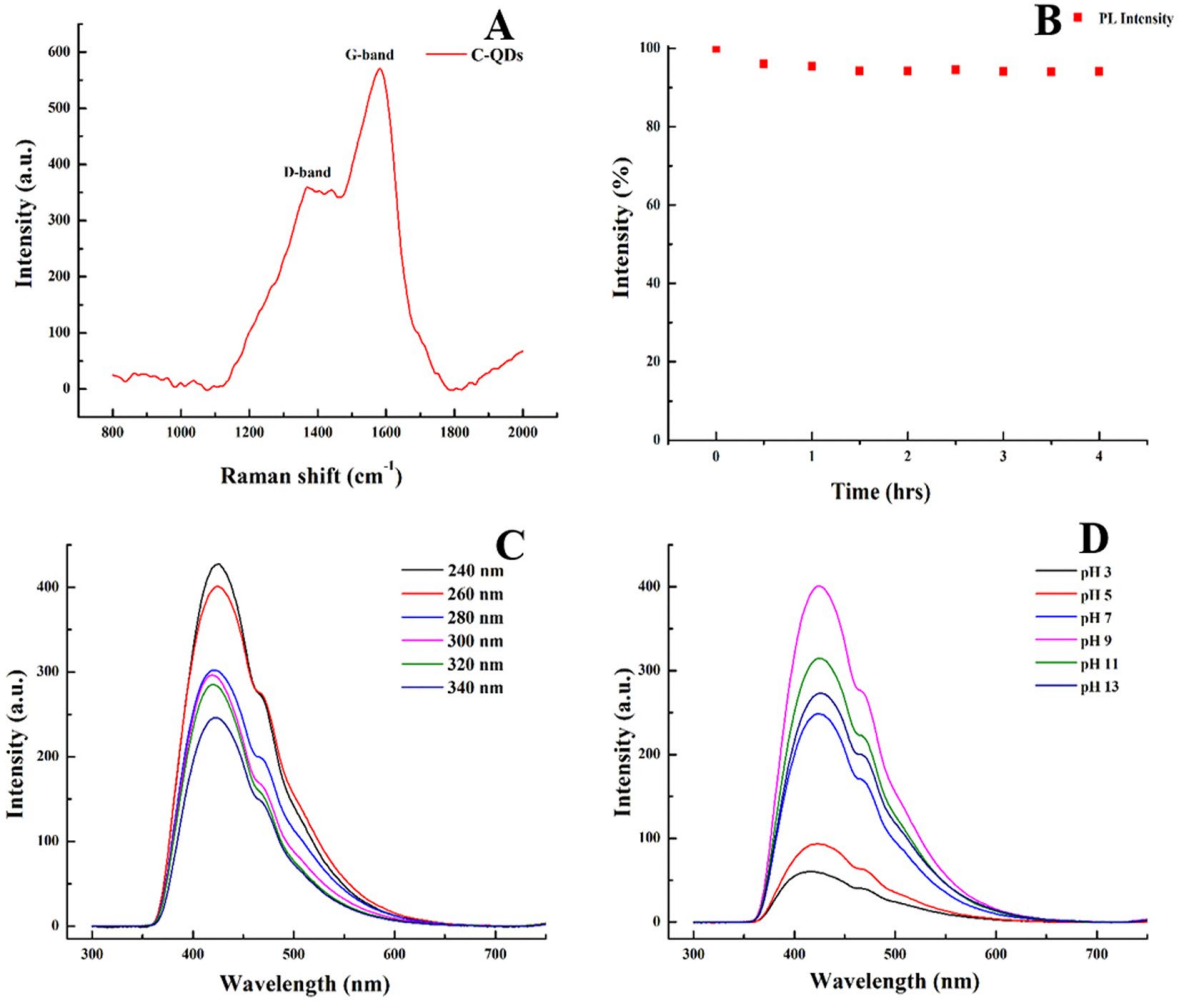
(**A)** Raman spectra of as-synthesized C-QDs (excitation wavelength 785 nm) shows G-band (1582 cm^−1^) and D-band (1371 cm^−1^), **(B)** Photoirradiation stability test shows that continuous irradiation from a xenon lamp of 150 W for 4 hours attribute almost no meaningful reduction in the PL intensity, **(C)** PL emission spectra of C-QDs excited at various energies shows PL of C-QDs was independent of the excitation energy (no redshift was found), **(D)** PL emission spectra of C-QDs at various pH (acidic to basic) shows PL of C-QDs was independent of the excitation energy (excitation wavelength 260 nm).

Raman spectrum of C-QDs recorded with 785 nm excitation wavelength is shown in Fig. 8A. Raman spectrum shows two distinct peaks at 1371 (D-band) and 1582 cm^−1^ (G-band), respectively. The G-band (sp^2^ hybridization) represents the amount of graphitization associated with the C-QDs and D-band (sp^3^ hybridization) represents the amount of defect and functionalization. The intensity ratio of the peak of the G band to the D band was found to be 1.58, which is higher then the C-QDs synthesized by other natural carbon sources^14,16,17,29^. The higher G/D ratio indicates that the synthesized C-QDs are composed of graphitic crystalline structure. HRTEM images (see Fig. 4B) show that the C-QDs have no amorphous structure, and the presence of D-band might be because of the functionalization of C-QDs.

The photoluminescence of C-QDs is stable under photoirradiation exposure, as shown in Fig. 8B. Continuous irradiation from a xenon lamp of 150 W for 4 hours attribute almost no meaningful reduction in the PL intensity, and that implies the superior photostability of as-synthesized C-QDs^9,34,35^. Unlike to the amorphous C-QDs, the thorough carbonization and better crystalline structure might be the reason for excellent photostability of as synthesis C-QDs^36^.

### Classical photoluminescence analysis of C-QDs

The quantum yield of as-synthesized C-QDs (excitation wavelength 260 nm) was calculated to be 9.5%. Adequate purification and single molecular C-QD tracking studies are necessary to precisely answer what may be the prominent reason for the PL mechanism in CDs^37^. PL mechanisms are constantly under debate; Z. Gan *et al*. have presented the possible PL mechanism involving excitation-dependent and excitation-independent PL^38^. The quantum confinement effect^17^, surface traps, the electronegativity of heteroatoms and synergistic models were purposed for the carbon nanostructures, including the C-QDs, exhibiting excitation-dependent PL^8,16,17,38^. On the contrary, surface engineering (surface state), altering the degree of carbonization and shape (hollow interiors) of C-QDs was a plausible explanation for excitation-independent PL^38^. Figure 8C shows the PL of C-QDs dispersed in water, the PL emission spectra of C-QDs was independent of the excitation wavelength and had a broad asymmetric peak ranged from 355 to 627 nm. The broad PL emission spectral range follows the C-QDs size distribution curve (the tail extending towards the higher wavelength). The shape of typical PL emission spectra possesses two peaks; (i) the main peak centered at 417 nm (FWHM 50 nm), and (ii) a shoulder peak at 470 nm (FWHM 90 nm).

The excitation independent C-QDs can be readily synthesized from the synthetic carbon precursor, and the quantum confinement was the reason sought for the PL explanation^2,10,34^. However, C-QDs synthesized from the natural carbon source in the present report is excitation independent as C-QDs shows no red-shift that indicates quantum confinement can be overruled (see the SI Fig. S6 for normalized spectra of C-QDs excitation at a different wavelength)^6,8,9,13,16,17,19–21,24,25^. Since there was no surface modification and as-synthesized C-QDs have a spherical shape (no hollow carbon structure was observed), the surface engineering and shape inspired excitation-independent PL mechanism can also be ruled out altogether^38^. However, an increase in the degree of carbonization can reduce the amount of defects and, in turn, surface states of C-QDs become more narrow and uniform^39^. Given the fact that the as synthesize C-QDs, having uniform size distribution, were synthesized at higher temperature 500 °C (3 hours), also the TEM and Raman results show that the as synthesize C-QDs have a high degree of carbonization. It is obvious to conclude that the C-QDs have a low amount of defects i.e., uniform surface states of the C-QDs.

The bandgap of CDs (carbon dots) decreases with an increase in degree of oxidation i.e., CDs with a lower degree of oxidation are more likely to be excitation-independent^40^. The CDs were containing multifunctional oxygen (C-C, C=O, C-O-C, C-O-H) inclined to be excitation-dependent^41^. As suggested by ref.^41^, excitation independent PL can be achieved by the tuning the C=C/C=O (higher is preferred) and C-O/C-O-C peak ratio (lower is preferred). XPS results show that as-synthesized C-QDs does not have multifunctional oxygen, the C=C/C=O peak ratio was higher, and C-QDs have a moderate degree of oxidation (<30%) and, these possibilities are the reason that emission of C-QDs to be excitation-independent. It is well known that there are π–π* and n–π* transitions in CDs, it is believed that the fluorescence mechanism in the present case is attributed to the surface states transition of C=O functional group (n–π*) attach onto the C-QDs and almost all excited electrons returned to the ground state via a radiation energy transfer^2,17,42–44^.

Recent studies have shown that the PL was originated from the fluorophores in CDs and CDs intrinsic structure, with little or no graphitic formation, was not contributing to the emission of CDs^45–48^. However, the chances of as-synthesized C-QDs have fluorophores are negligible. With regards to the fluorophores, there are two possibilities; fluorophores are either present independently with the CDs^47^ or attached on the surface of CDs^45^. The quantum yield (QY) of CDs (absence of fluorophores) was reported far lower (around 7%) compared to the fluorophores-functionalized CDs, which was quite high (usually more than 50%)^45^. As synthesized C-QDs in the present report has a quantum yield of 9.5%, the QY of our C-QDs aligned with ref.^45^, where the CDs (absence of fluorophores) had lower QY in comparison to fluorophores-functionalized CDs. Therefore, it is concluded that as-synthesized C-QDs do not have fluorophores attached on the surface of C-QDs. Moreover, fluorophores are formed at mild reaction conditions (short-reaction time and low temperature ~200 °C) and have a hydrodynamic diameter ~1 nm^47^. Given the fact that the reaction temperature in the present report was 500 °C (3 hours), the chances of forming the fluorophores are less likely than carbonization. In addition, the TLC results shows that as-synthesized C-QDs, after the dialysis, has only a single fraction (see the SI Fig. S7) and the DLS measurements also shows no peak having hydrodynamic diameter around 1.0 nm that successfully validate the assumption that fluorophores don’t exist independently in the purified C-QDs^47^. Single fraction in TLC results shows that as synthesized C-QDs have only one kind of species and as synthesized C-QDs have high purity^49^.

The photoluminescence (PL) of the C-QDs is sensitive to the external parameters such as pH and the type of the solvent that C-QDs are dispersed. Photoluminescence of C-QDs disperse in the water at different pH ranging from 3 to 13 (acidic to basic) is shown in Fig. 8D. The PL of C-QDs has a broad emission peak position, centered at 419 nm, is independent of the pH^5–11^. However, changing the pH from acidic to basic resulted in a gradual increase in the PL intensity of C-QDs, similar trends observed earlier^16^. The highest PL intensity of C-QDs was found to be in the range of pH (9–11), i.e., above the neural conditions. Unlike to the excitation-dependent C-QDs, the emission PL peak of C-QDs in strong acidic (pH = 3) and basic media (pH = 13) shift towards the shorter and longer wavelength, respectively, however, the wavelength shift of as-synthesized C-QDs is not very significant (see the SI Fig. S8)^16^. We believe that in case of wavelength-dependent C-QDs (multiple surface states dominate the PL) the surface states were majorly capped by the availability of the numerous functional groups and so by changing the pH from acidic to basic the different set of surface states get activated. Whereas for the wavelength-independent C-QDs in the present case, the availability of the surface states has a limited role to play given the moderate amount of functionalization and well-crystalline C-QDs structure. Photoluminescence of C-QDs shows strong fluorescence, robust photo- and chemical stability.

### ML-assisted photoluminescence analysis of C-QDs

For the pH stability study, most of the C-QD’s PL investigations done so far is at a given particular excitation, often chosen randomly, and completely ignore the other possible excitation. It is because while varying the pH and excitation simultaneously for a particular C-QDs sample, it will generate a large data set of PL measurements and at times it is difficult to manage and analyze the extensive data set with classical analytical techniques. However, examining the C-QDs at the different set of pH for a wide range of excitation can, possibly, provide much valuable information than the conventional analysis techniques.

We used the machine learning techniques (ML) such as PCA, MCR-ALS, sparse-NMF to handle large PL dataset. Going forward, the PL data collected at multiple excitations and pH will be addressed as integrated PL dataset. PCA was employed to choose the excitation wavelength with objectivity for chemical stability (pH) test, i.e., excitation wavelength that explains the maximum variance in the PL. Also, using the ML feature extraction techniques such as MCR-ALS and sparse-NMF analysis the following crucial issue was addressed; a typical PL spectrum of as-synthesized C-QDs has a very broad and asymmetric peak, and a change in the excitation or pH can activate the PL from multiple surface states depending on the functionalization. Moreover, it is possible that the C-QDs’ PL peak might be the linear combination of two or more asymmetric and/or symmetric peaks which directly means the existence of two or more different kind of PL phenomenon^8,17^. MCR-ALS and sparse NMF techniques are used to investigate whether the PL of as-synthesized C-QDs is from multiple surface states functionalization or a singleton energy state.

### PCA analysis

**Figure 9:**
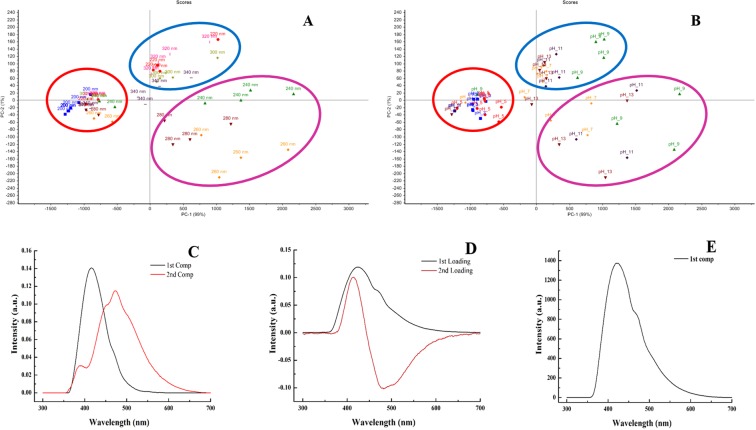
**(A)** PCR score matrix of integrated PL dataset (Labels- wavelength), **(B)** PCR score matrix of integrated PL dataset (Labels- pH). PCA analysis has successfully clustered the Integrated PL dataset into three groups: (i) excitation at 200 nm (pH 3, 5, 7, 9, 11 and 13), (2) excitation at 300, 320 and 340 nm (pH 9, 11, 13), and (3) excitation 240, 260 and 280 nm (pH 7, 9, 11, 13) marked with red, blue and maroon color ellipse, respectively, **(C)** MCR-ALS analysis shows two spectral signatures (centered at 416.2 and 475.3 nm) extracted from integrated PL dataset have considerable peak overlapping, **(D)** PCA analysis also depicts the presence of two distinct feature vectors. However, the second negative loading makes the data interpretation more complicated, **(E)** NMF-ARD-SO analysis of integrated PL dataset results into a single spectral peak centered at 424.7 nm, resembled the original PL spectrum.

There have been many reports where PCA was utilized to extract the prominent feature vectors from a large data set (1-D, 2-D, and 3-D spectral analysis), however, for C-QDs’ PL data analysis PCA has not been used. PCA is a data compression process; unknown orthogonal spectral vectors are extracted, and similar PL data is group together (see more details for PCA in SI)^50^. The first two principal components (PCs), extracted from the integrated PL dataset of as-synthesized C-QDs, together explained the entire dataset; given the first and second PCs contribute 99.0 and 1.0%, respectively. PCA score matrix of integrated PL dataset is shown in Fig. 9A,B, respectively. For the sake of clarity, the same PCA score matrix is shown with different text labels separately; pH and excitation. PCA analysis has clustered the integrated PL data set into three groups: (i) excitation at 200 nm (for all pH 3 to 13) & 220 to 340 nm (for acidic pH 3 & 5), (ii) excitation at 300, 320 and 340 nm (for basic pH 9, 11, 13), and (iii) excitation 240, 260 and 280 nm (for basic pH 7, 9, 11, 13) marked with red, blue and maroon color ellipse, respectively. It is evident from the first subgroup (red color) that at acidic pH, irrespective of the different excitation, the spectra fall into one group, there is no noticeable peak shift (see the SI Fig. S9). Conversely, for the second and third subgroups the PL peak is marginally shifted towards higher wavelength at basic pH condition, however, the shift is not very significant; less than 1 nm (see the SI Figs S10 and S11).

First and second group is distributed over the small region, however, interestingly, the third group is spread over a large area, it might be because of the fact that the maximum PL intensity was also observed at 260 nm excitation and a very minute change in pH around 240–280 nm excitation results into evolution of new spectral features. Since, PCA analysis has successfully categories the integrated PL data into three groups, the excitation wavelength that shows the similar or dissimilar features can be chosen with objectivity. It is evident that the group marked with maroon-color ellipse has large variance, and it is the same excitation wavelength (260 nm) that should be chosen for PL studies to investigate the possible PL mechanism. Although, PCA has successfully categories the integrated PL data-set, nevertheless, it fails to provide any information on the typical asymmetric PL peak of as-synthesized C-QDs.

### MCR-ALS analysis

The standard peak feature extraction procedure becomes inefficient to understand whether the PL spectrum of C-QDs is a single peak or convolution of multiple peaks. The MCR-ALS analysis helps to extracts the pure spectral profile of a PL spectrum from the integrated PL data-set of C-QDs^26^. Figure 9C shows the MCR-ALS results, integrated PL data-set was resolved into two spectral peaks centered at 416.2 and 475.3 nm, respectively, indicating that the typical PL spectrum is a linear combination of two distinct peaks (feature vectors). Although MCR-ALS was able to resolve the PL spectra into two distinct peaks successfully, nevertheless, the newly resolved peaks overlap, considerably, on each other to such an extent that it is difficult to assign them to two different energy states. The PCA analysis of integrated PL data-set also results in two distinct feature vectors except that the second peak for loading vector is negative that makes the data interpretation more complicated (Fig. 9D). There are two fundamental issues using MCR-ALS analysis; the first issue is more critical to our PL data-set, i.e., the MCR-ALS extracted peaks are overlapped, so hard to assign them to a particular energy state. The second issue is, ones have to manually select the number of components that are to be extracted, and ones are free to choose may two, three, four or any number of component and MCR-ALS will still fit all the data. The non-negative matrix factorization (NMF) technique with a penalty term auto relevance determination (ARD) can select the number of components to be fitted automatically, and by imposing the soft orthogonal constraint (SO), the overlap peaks can be resolved distinctly^51^.

### NMF-ARD analysis

The NMF-ARD-SO analysis of integrated data-set results into single spectral peak centered at 424.7 nm, resembled the original PL spectrum (see Fig. 9E). Even imposing the hard penalty constraints has not helped and only a single peak could be extracted. The absence of any new peak essentially shed light on the fact that a single broad peak is because of the intrinsic nature of the as synthesize C-QDs, i.e., possibly, single energy transition state exists. Although, we have substantial functionalization on the surface of C-QDs, nonetheless, the photoluminescence is not because of the multiple surface states and the possibility of the existence of multiple energy states can be ruled out. Our results show that given the advantage of priory and orthogonal constrained to ascertain the origin of the PL of C-QDs NMF-ARD-SO analysis has merits over the MCR-ALS and PCA.

## Conclusion

We have synthesized mono-dispersed C-QDs via single-step thermal decomposition process using eco-friendly, high abundance, and very economical fennel seeds (*Foeniculum vulgare*). As synthesize C-QDs were well graphitized and possess excellent colloidal solubility, photo-stability, and environmental stability (pH). The C-QDs show excellent PL activity and excitation-independent emission. Machine learning techniques (ML) such as PCA, MCR-ALS, NMF-ARD-SO are useful in handling the large PL data-set and helped to ascertain the origin of the PL mechanism of as-synthesized C-QDs. PCA helped to choose the best excitation wavelength with objectivity for PL analysis. Nevertheless, we recommend the use of NMF-ARD-SO algorithm to analyze the PL mechanism because of its advantage over other ML algorithms. Synthesis of excitation independent C-QDs is easy to employ and can be scaled up for mass production. Such C-QDs is unique and may find lots of applications in bio-sensing, cellular imaging, solar cell, and sensors.

## Supplementary information

Supplementary information
