# Supplementary material for: Synthesis and characterization of Mono-disperse Carbon Quantum Dots from Fennel Seeds: Photoluminescence analysis using Machine Learning
Source: Sci Rep. 2019 Sep 30;9:14004. doi: 10.1038/s41598-019-50397-5 (PMC6769153; doi:10.1038/s41598-019-50397-5)
Supplement: Supplementary file 1 — Supplementary information [file 41598_2019_50397_MOESM1_ESM.pdf]

Supporting Information

Synthesis and characterization of Mono-disperse Carbon Quantum Dots from Fennel Seeds: Photoluminescence analysis using Machine Learning

Akansha Dager[1], Takashi Uchida[2,*], Toru Maekawa[2], Masaru Tachibana[1].

[1]Graduate School of Nanobioscience, Yokohama City University, 22-2 Seto, Kanazawa-Ku, Yokohama, 236-0027, Japan.

[2]Bio-Nano Electronics Research Centre, Toyo University, 2100 Kujirai, Kawagoe, Saitama 350-8585, Japan.

[*]Present address: Silicone-Electronics Materials Research Center, Shin-Etsu Chemical Co., Ltd., 1-10 Hitomi, Matsuida-machi, Annaka-shi, Gunma, 379-0224, Japan.

Contents

1. Data structure for the integrated PL measurements
Photoluminescence Data structure

2. ML techniques for PL Data analysis
(i) Principal Component Analysis (PCA)
(ii) Multivariate Curve Resolution- Alternating Least Squares (MCR-ALS) analysis
(iii) Sparse Nonnegative Matrix Factorization (NMF) analysis

3. Distribution of the diameters of as synthesized C-QDs.
Figure S1

4. Higher resolution TEM image of C-QDs.
Figure S2

5. Point beam EDS spectra of as synthesized C-QDs.
Figure S3

6. Colloidal stability of as synthesized C-QDs.
Figure S4

7. Optimization of the pyrolysis process parameters for the synthesis of C-QDs.
Figure S5

8. XPS analysis of as-synthesized C-QDs
Table S1- Wide scan of as-synthesized C-QDs
Table S2- Peak fitting results obtained after deconvolution of carbon peak
Table S3- Peak fitting results obtained after deconvolution of oxygen peak

9. Normalized PL emission spectra of C-QDs.
Figure S6

10. Thin Layer chromatography of C-QDs.
Figure S7

11. Effect of pH on PL of as synthesized C-QDs (Environmental stability).
Figure S8

12. PCA analysis of as synthesized C-QDs.
Figure S9 – group (I)
Figure S10 – group (II)
Figure S11 – group (III)

13. References

1. Data structure for the integrated PL measurements

Photoluminescence Data structure: PL data was acquired using the excitation at 200, 220, 240, 260, 280, 300, 320 and 340 nm, respectively for the following pH 3, 5, 7, 9, 11, 13. One spectrum, i.e., intensity vs. wavelength is stored in one-dimensional vector of length (n, the number of data point acquired in spectral range 1: n). Consequently, all the spectra was put together to form the two dimensional (2D) matrix; i.e., D (48 x n) of size (samples x n) containing the entire PL data set. Each row in the 2D data matrix represents one spectrum. Since, the data have good signal to noise ratio, no additional data processing was done.

2. ML techniques for PL Data analysis

(i) Principal Component Analysis (PCA): Principal component analysis entrust on the fact that the wavelength is the variable space (feature space) and it is interrelated, i.e. correlation exist. Each principal component (PC) is a linear combination of all the original feature variables. The first PC contributes the maximum variance in the dataset, and second PC is uncorrelated (orthogonal) to the first PC and account for the next possible variance and so on. Although, there can be as much as number of PC as the feature variable, however, the first few PC are of great importance and remaining have noise or minimal information and if can be discarded have not much impact on the given dataset. The PCA analysis of D matrix resulted to two-sub matrix of S (score) and L (loading) using NIPAL algorithm [1].

$D = CS^T$

where D, S^T, C and E are the data set (PL spectra), loading (spectral profile), and score matrix, respectively.

(ii) Multivariate Curve Resolution- Alternating Least Squares (MCR-ALS): MCR is useful for resolving the spectroscopic data featuring peaks that contain relevant spectral profile (S^T), even estimating the concentration (C) from mixture spectra [2]. Since, the PL spectrum of as synthesized C-QDs is quite broad, MCR-ALS was performed to extract the possibility of the other peaks. The MCR-ALS with non-negative constrained was performed using MCR-ALS tool [2]. The major hindrance for the MCR-ALS is that ones must know the expected number of components in advance to optimize the MCR-ALS regression. An incorrect choice can lead to overestimation (inclusion of noise) and underestimation (loss of information). PCA was used to choose the number of components with objectivity. The number of components using PCA was found to be two. MCR-ALS was optimized for the extraction of two spectral component using

$D = CS^T + E$

where D, S^T, C and E are the data set (PL spectra), loading (spectral profile), score matrix and E (residual), respectively.

(iii) Sparse Nonnegative Matrix Factorization (NMF): For the sparse NMF, a recently developed technique by Shiga et Al. (hierarchical alternating least squares (HALS) algorithm) was used [3]. Unlike to the MCR-ALS, spares NMF automatically optimize the number of components with the choice of automatic relevance determination penalty term “prior” (ARD) along with the desired orthogonal constrained (NMF-ARD-SO). Soft orthogonal constraint with ARD prior, overcome the limitation of MCR-ALS; i.e. no knowledge required for the number of component selection and overlap peak if any can be resolve with appropriate soft or hard orthogonal constraint. Sparse NMF was optimized for a soft orthogonal constrained (0.1) with exponential prior [3].

3. Distribution of the diameters of as synthesized C-QDs.

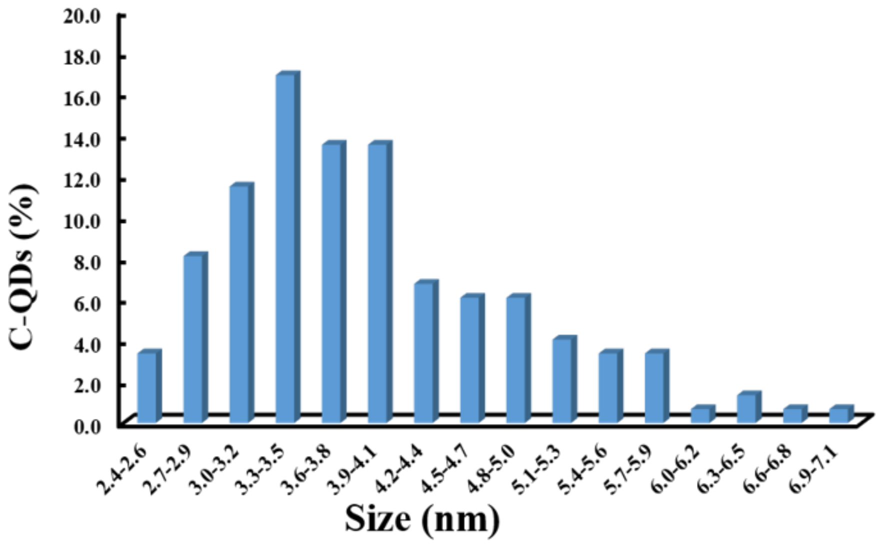

Figure S1. Distribution of the diameters of as synthesized C-QDs. The average diameter and standard deviation of C-QDs were 3.90 and 0.91 nm, respectively. More than 97 % of C-QDs have diameter in the range of 2.4 – 5.9 nm.

4. Higher resolution TEM image of C-QDs.

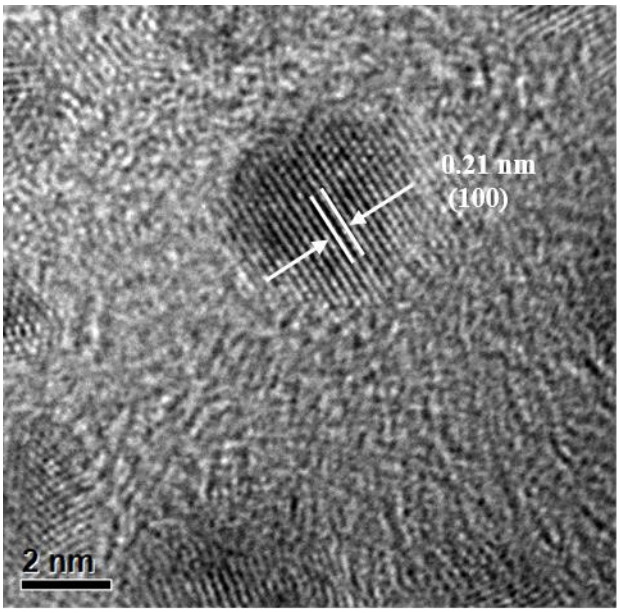

Figure S2. Higher resolution TEM image of C-QDs shows that C-QDs have crystalline structure and the distance between the lattices fringes is 0.21 nm assigned to (100) plane.

5. Point beam EDS spectra of as synthesized C-QDs.

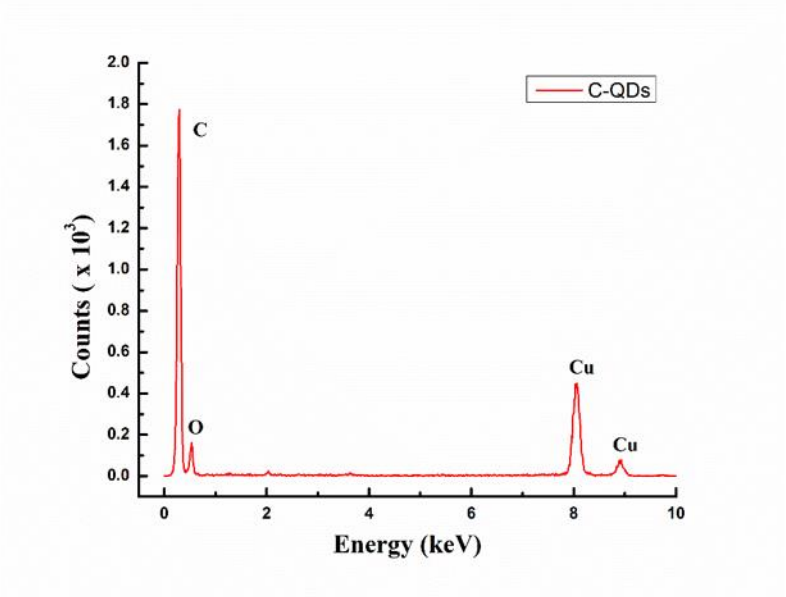

Figure S3. Point beam EDS spectra of as synthesized C-QDs. No other trace element was detected other than carbon and oxygen.

6. Colloidal stability of as synthesized C-QDs.

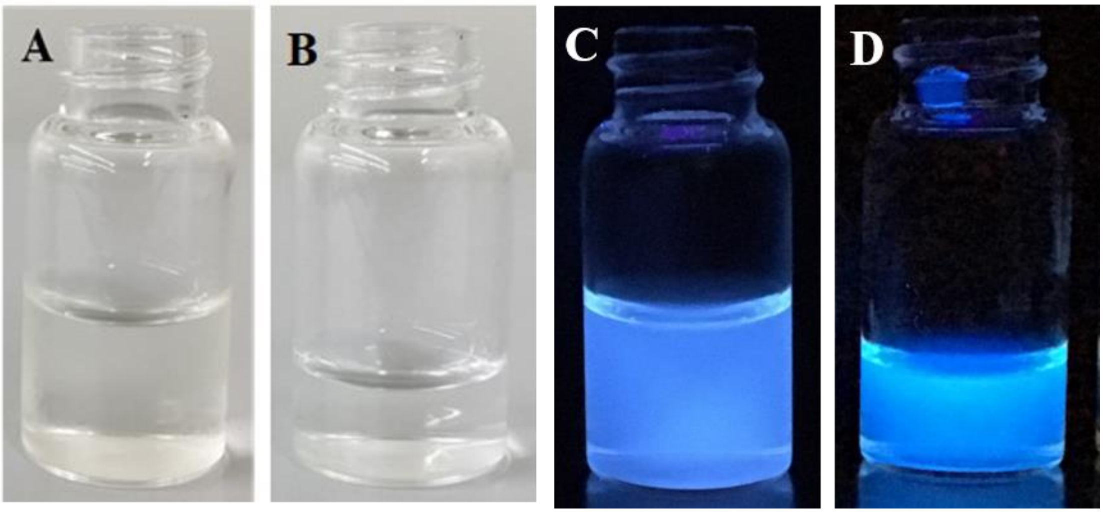

Figure S4. C-QDs shows no sign of turbidity even after 15 months of storage in ambient condition. (A) Optical image of Freshly synthesized C-QDs dispersed in water under daylight exposure, (B) Optical image of C-QDs dispersed in water under daylight exposure (after 15 months storage), (C) Optical image of Freshly synthesized C-QDs dispersed in water under UV exposure, (D) Optical image of C-QDs dispersed in water under UV exposure (after 15 months storage).

7. Optimization of the pyrolysis process parameters for the synthesis of C-QDs.

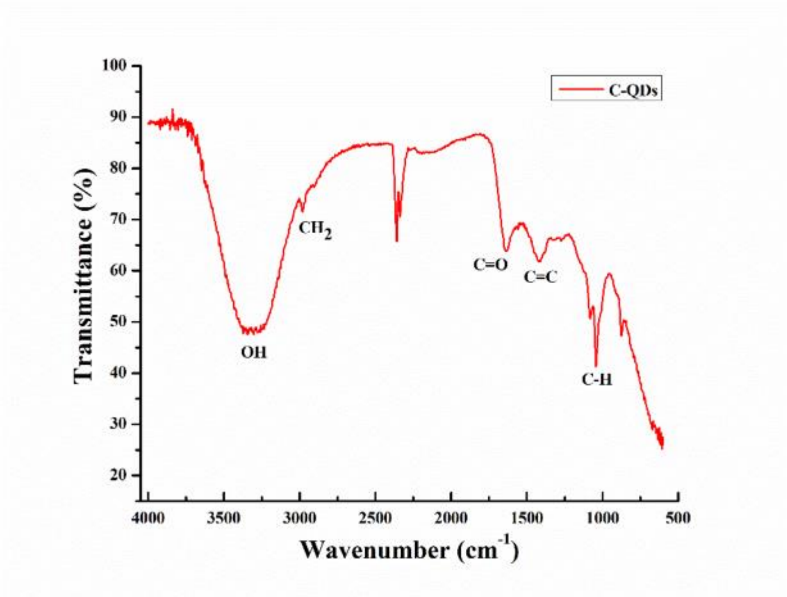

Figure S5. Symmetric and Asymmetric peaks of CH_2 as an indicator to optimize the pyrolysis process parameters (sensor to distinguish the insufficient carbonization). FTIR spectra taken from a typical C-QDs synthesis trial at 300 ºC for 3 hours. The presence of CH_2 peaks in FTIR at 2921 and 2851 cm^{-1} shows incomplete pyrolysis of ground fennel powder.

8. XPS analysis of as-synthesized C-QDs

Table S1. XPS analysis of as-synthesized C-QDs

**S.No.**	**Element**	**Peak position BE (eV)**	**Atomic concentration (%)**
1	C 1s	285	71.2
2	O 1s	531	28.8

Table S2. Peak fitting results obtained after deconvolution of carbon peak

**Peak**	**Assignment**	**Peak position BE (ev)**	**FWHM (eV)**	**Atomic concentration (%)**
C 1s	C-sp 2	284.60	0.98	70.2
C 1s	C-O	286.16	1.17	20.6
C 1s	C=O	287.20	1.89	9.20

Table S3. Peak fitting results obtained after deconvolution of oxygen peak

**Peak**	**Assignment**	**Peak position BE (ev)**	**FWHM (eV)**	**Atomic concentration (%)**
O 1s	Phy absorbed O_2	529.13	1.80	11.5
O 1s	OH/C=O, O-C=O	531.10	1.73	88.5

9. Normalized PL emission spectra of C-QDs.

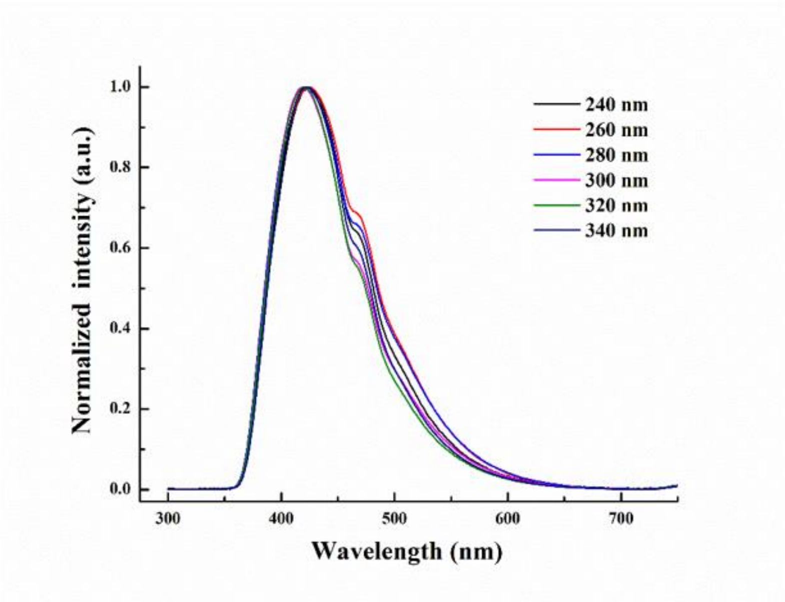

Figure S6. Normalized PL emission spectra of C-QDs excited at various wavelength (240 – 340 nm). PL emission spectrum of C-QDs was independent to the excitation wavelength (no redshift was observed).

10. Thin Layer chromatography of C-QDs.

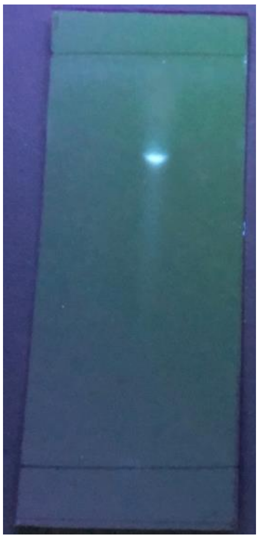

Figure S7. Thin layer chromatography (TLC) of as synthesized C-QDs. TLC shows a single luminescent band.

11. Effect of pH on PL of as synthesized C-QDs (Environmental stability).

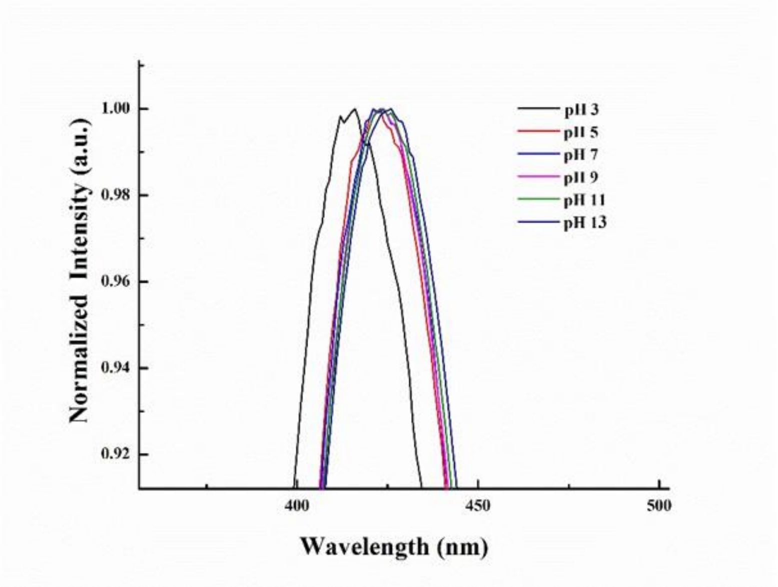

Figure S8. Effect of pH on PL of as synthesized C-QDs (acidic to basic pH). PL of carbon quantum dots (Normalized) in strong acidic (pH 3) and basic media (pH 13) shifts towards the shorter and longer wavelength, respectively. However, the shift is not very significant as can be seen in the zoomed image of PL (excited wavelength 260 nm).

12. PCA analysis of as synthesized C-QDs.

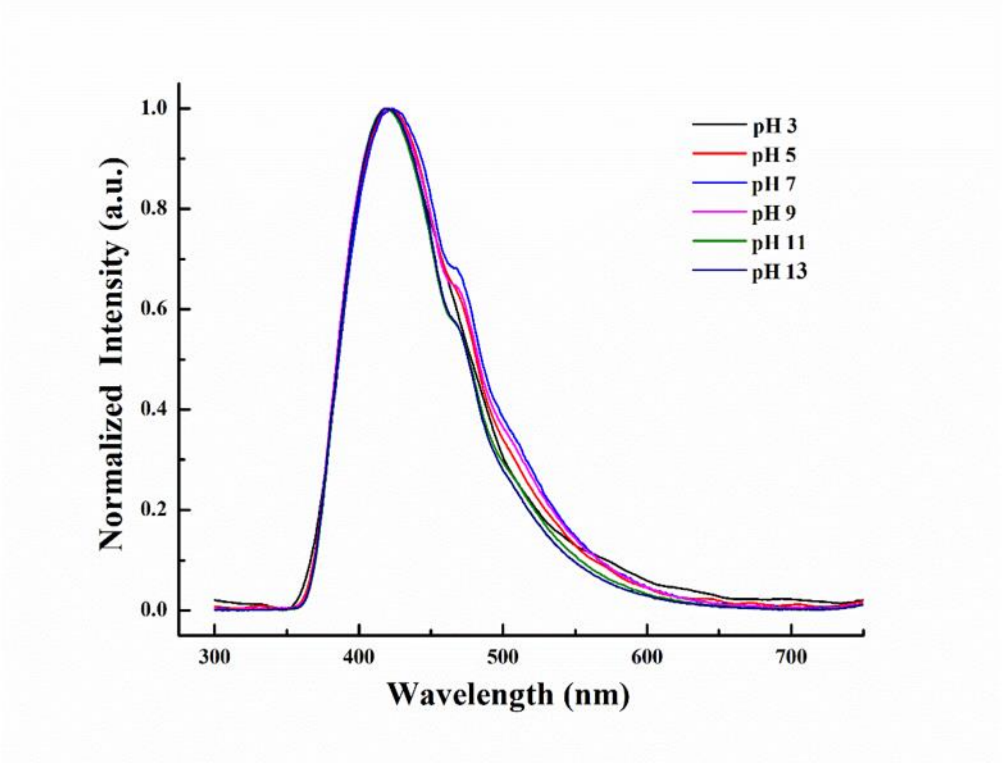

Figure S9. PCA classified group-I (labeled with red color) comprising the normalized PL spectrum at pH 3,5,7,9,11 and 13, respectively (excitation wavelength 200 nm).

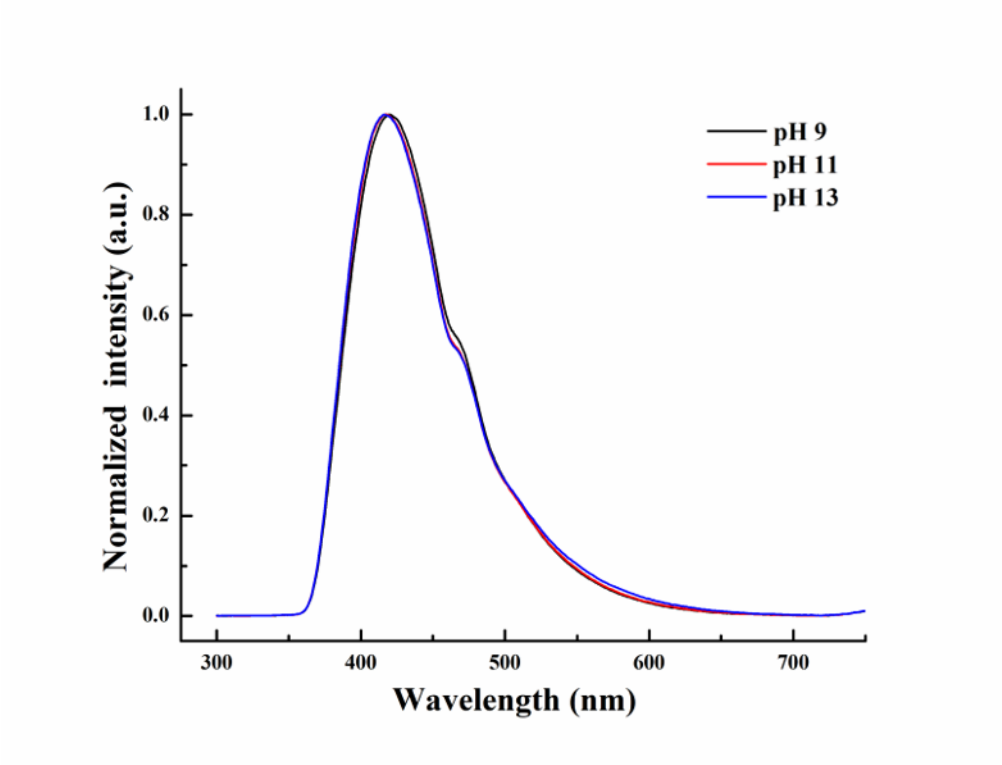

Figure S10. PCA classified group-II (labeled with blue color) comprising the normalized PL spectrum at pH 9, 11 and 13, respectively (excitation wavelength 320 nm).

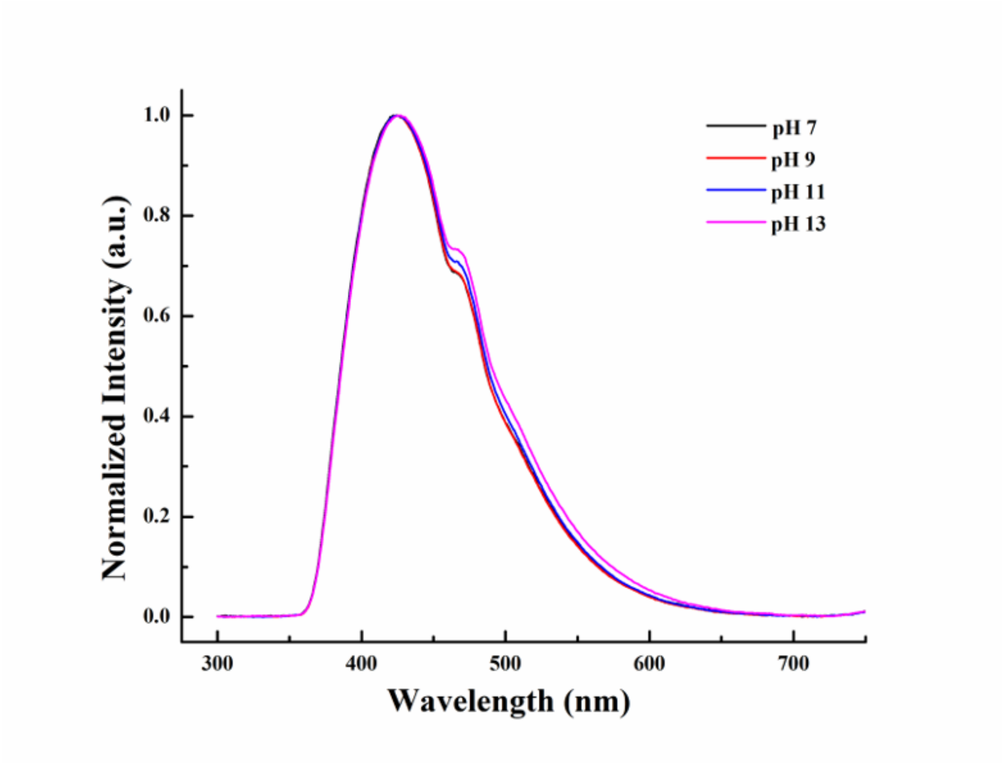

Figure S11. PCA classified group-III (labeled with maroon color) comprising the normalized PL spectrum at pH 7, 9, 11 and 13, respectively (excitation wavelength 260 nm).

13. References:

1. Judith Felten, Hardy Hall, Joaquim Jaumot, Romà Tauler, Anna de Juan and András GorzsásVibrational, Spectroscopic image analysis of biological material using multivariate curve resolution–alternating least squares (MCR-ALS), *Nature Protocols*.,**10**, 217–240 (2015).

2. O.A. Maslova, G. Guimbretière, M.R. Ammar, L. Desgranges, C. Jégou, A. Canizarès, P. Simon, Raman imaging and principal component analysis-based data processing on uranium oxide ceramics, *Mater. Charact*., **129**, 260–269, (2017).

3. M. Shiga, K. Tatsumi, S. Muto, K. Tsuda, Y. Yamamoto, T. Mori, T. Tanji, Sparse modeling of EELS and EDX spectral imaging data by nonnegative matrix factorization, *Ultramicroscopy*, **170,** 43-59, (2016).
